# The 4 U’s Rule of Fibromyalgia: A Proposed Model for Fatigue in a Sample of Women with Fibromyalgia: A Qualitative Study

**DOI:** 10.3390/ijerph17176224

**Published:** 2020-08-27

**Authors:** Lilian Velasco-Furlong, Lorena Gutiérrez-Hermoso, Beatriz Mateos-Pintado, Daniel Garvi-de Castro, Sheila Blanco-Rico, Lucía Sanromán-Canelada, Sofía López-Roig, María Ángeles Pastor-Mira, Cecilia Peñacoba-Puente

**Affiliations:** 1 Department of Psychology of Universidad Rey Juan Carlos, Avenida Atenas s/n, 28922 Alcorcón, Spain; lilian.velasco@urjc.es (L.V.-F.); lorena.gutierrezh@urjc.es (L.G.-H.); mateos.pintado.beatriz@gmail.com (B.M.-P.); dangarvi@ucm.es (D.G.-d.C.); sheila.blanco.rico@urjc.es (S.B.-R.); lucialogrosan@gmail.com (L.S.-C.); 2 Department of Behavioural Sciences and Health of Universidad Miguel Hernández, Avinguda de la Universitat d’Elx s/n, 03202 Elche, Spain; slroig@umh.es (S.L.-R.); mapastor@umh.es (M.Á.P.-M.)

**Keywords:** fibromyalgia syndrome, fatigue, focus groups, qualitative research, hybrid approach, narrative analysis

## Abstract

Although fatigue usually goes unnoticed, it is a symptom that poses great challenges to patients with fibromyalgia and is a strong limitation. The aim of this study is to identify and describe the variables involved in fatigue in nine different situations of the Goal Pursuit Questionnaire (GPQ) that may occur in the daily lives of women with fibromyalgia, according to an ABC (Antecedents–Behaviors–Consequences) model. This study followed a qualitative descriptive research method and a deductive–inductive hybrid approach based on a phenomenological paradigm. Twenty-six women with fibromyalgia participated in focus group discussions between February and March of 2018. Thematic content analysis was carried out from transcribed verbatim interviews. We identified nine major themes that emerged from the participants’ conversations: self-imposed duties, muscle fatigue, overwhelming feeling of tiredness, difficulty thinking, difficulty concentrating, negative emotions, lifestyle changes, affected everyday activities, and lack of motivation for daily activities and social interactions. We conclude that the ABC model allowed certain elements to emerge regarding the fatigue experience, highlighting its importance as a symptom in fibromyalgia. This additional analysis of the ABC model showed that fatigue can be described through the 4 U’s Rule, which is integrated by these four adjectives: (1) Unpredictable, (2) Uncontrollable, (3) Unseen, and (4) Unintelligible. Identifying these characteristics can contribute to a better understanding of fibromyalgia in addition to better treatment for these patients.

## 1. Introduction

Fibromyalgia (FM) is a chronic disorder characterized by widespread pain and tenderness [1]. In 2010, the American College of Rheumatology (ACR) [2] stated that a patient has to meet the following conditions to be diagnosed with fibromyalgia: [1] Widespread Pain Index (WPI) ≥7 and Symptom Severity Scale (SSS) score ≥5 or WPI 3–6 and SSS Scale Score ≥9; [2] symptoms have to have been present at a similar level for at least 3 months; and [3] the patient should not have a disorder that would otherwise explain the pain. In 2016, the cut-off points for the WPI scale measuring widespread pain changed, being currently set at 4–6 [3]. The worldwide fibromyalgia average prevalence is estimated 2.7% in the general population, with a greater prevalence among women (4.1%) than men (1.4%) [4]. The ACR criteria include fatigue as a diagnostic symptom for fibromyalgia alongside sleep disturbances, memory problems, and difficulty concentrating [2,3]. Therefore, these criteria highlight that pain is not the only symptom of fibromyalgia, and furthermore, previous studies have identified other bothersome symptoms that have a greater impact on health status (physical and psychosocial impact) [5,6]. Among all these symptoms, fatigue ranked as the second most important domain to be measured, and it was considered by patients as the third most important symptom after pain and sleep disturbances [6].

Piper [7] defined fatigue in fibromyalgia as “an unusual, abnormal or excessive whole-body tiredness, disproportionate to or unrelated to activity or exertion”. Fatigue can be differentiated from simple tiredness because the former is not explained by life strain and cannot be eased with the usual management strategies, such as sleeping or resting [8]. Experts in this matter suggest that fatigue in fibromyalgia patients may be caused by a dysfunction in the hypothalamus–pituitary–adrenal axis (HPA). Accordingly, research has shown lower cortisol levels (hypocortisolism) in these patients [9] as well as a deficiency of serotonin, melatonin, and cytokines, which are all fully regulated by the circadian rhythm [10]. Furthermore, stress has been suggested to be one of the causes of this HPA dysfunction; therefore, fatigue and lower quality of sleep could be explained as a specific result of deficit in cortisol and melatonin.

Although discovering the origins of fatigue is crucial, it is equally important to understand how it affects fibromyalgia patients. Some characteristics of fatigue in fibromyalgia include overwhelming feelings of tiredness that are not relieved by resting or sleeping, and are not proportional to the effort exerted; feelings of weakness or heaviness; difficulty in getting motivated; and cognitive difficulties. All these traits of fatigue have been integrated in Humphrey’s conceptual model of fatigue in fibromyalgia [11]. In their model, it was demonstrated that in addition to pain, fatigue was an important symptom for individuals with FM. Specifically, it was described as one of the worst symptoms of FM and was seen as a constant presence that required patients to pace the activities in their lives. The authors distinguish FM fatigue from normal tiredness from a patient’s perspective. This model is currently being used to support the development of a new patient-reported outcome for fibromyalgia fatigue [11]. On the other hand, other authors have suggested that fatigue also affects social relationships due to lack of understanding from significant others and unpredictability [8,12]. In addition, whilst women with fibromyalgia describe themselves as vigorous, caring, active, with high standards, and with a busy daily schedule filled with chores, family care, and professional work, they portray fatigue as intrusive and impossible to ignore [13]. Furthermore, patients with fibromyalgia find their own illness emotionally distressing and difficult to understand, consequently increasing their anxiety, desperation, and catastrophic thinking [14].

In order to sum up these findings, and as an addition to prior research, we propose a tentative model of antecedents (A)—that contribute to fibromyalgia, behaviors (B)—fibromyalgia traits divided into biological or cognitive–emotional, and consequences (C)—affected vital spheres of patients of fatigue in fibromyalgia. We based the ABC structure on science-based approaches of behavioral assessment, functional analysis, and clinical case formulation [15,16,17,18]. We used the guiding principle in psychological assessment to capture information to identify the multiple variables that can be influencing fatigue in FM patients. Identifying the antecedents or activating events, the behaviors (using two forms of responses) and consequences may help us to understand the elements that trigger fatigue. The great precision of ABC analyses allowed us to capture strategies for understanding the problem and to specify treatment goals [19,20].

Our aim in this study has been to identify and describe the variables involved in fatigue in nine different situations of the Goal Pursuit Questionnaire (GPQ) [21] that may occur in the daily lives of women with fibromyalgia according to the ABC model.

## 2. Materials and Methods

The Consolidated Criteria for Reporting Qualitative Studies (COREQ) [22] and the Standards for Reporting Qualitative Research (SRQR) [23] were integrated in the reporting of the methods and findings.

### 2.1. Design

A descriptive qualitative study was conducted based on a phenomenological paradigm [24,25]. This approach is based on the life experiences of individuals within their experiential world or ‘life-world’, describing how individuals make sense of the world and their subjective experience [26]. Moreover, this study followed a deductive–inductive hybrid approach, relying on the subjective experiences elicited in focus groups of women with fibromyalgia. Previous work has addressed the experience of FM in female patients [27]. The focus groups for this study were created as part of a larger study meant for the adaptation and validation of the Goal Pursuit Questionnaire (GPQ) by P.A. Karsdorp in Spanish women with fibromyalgia [21,28]. Specifically, for this qualitative analysis, women had to identify and describe the variables involved in fatigue in nine different situations that may occur in their daily lives.

### 2.2. Research Team

In accordance with previous work [27], and considering the theoretical framework and the team’s experience with reported symptoms among women with FM [29], the researchers set up three briefing sessions in which they expressed their motivation for the research. First, we wanted to gain insight into fatigue through the first-hand experience of women with fibromyalgia, which is, along with pain, the most disturbing symptom. Second, we wanted to describe and understand patients’ points of view regarding fatigue in nine different situations of the GPQ that may occur in their daily lives. The three sessions were guided by researchers who were experts in both qualitative design and research in chronic pain (SLR, MPM, CPP). Afterwards, three expert psychologists, with experience conducting groups, moderated the focus groups (LVF, SBR, LSC); two assistants were also present, taking notes (LGH, BMP).

### 2.3. Setting and Patients

The total sample consisted of 26 women with fibromyalgia living in Madrid, Spain. Participants were heterogeneous regarding age, marriage status, education, and professional background. The sample was obtained through a bigger Spanish fibromyalgia association (AFIBROM) located in the urban setting of Madrid. Phone calls were used to contact the aforementioned sample and arrange a date for the focus groups that were held in the Rey Juan Carlos University in Madrid, Spain. The entry criteria were established as being female, aged 18–70 years old, and who had received a diagnosis of fibromyalgia [30] by rheumatologists or primary care physicians. Diagnostic criteria were those established by the Spanish Ministry of Health, which follow the 1990 guidelines of the American College of Rheumatology. These are still used over the 2010 criteria, as it has been stated by the said Ministry that only the former guidelines are accepted by the scientific community, whilst the latter raise debate among the researchers of this field [31]. Exclusion criteria included the existence of concomitant rheumatic disorders, such as rheumatoid arthritis, systemic lupus erythematosus, Hashimoto’s disease, Sjogren’s syndrome, scleroderma, and reflex sympathetic dystrophy; and the existence of psychotic disorders, bipolar disorder, or other serious psychiatric conditions. Criterion sampling was used [32] and when the researchers achieved enough samples for three focus groups, data collection ended.

### 2.4. Recruitment

The researchers contacted the possible participants that belonged to a fibromyalgia association (AFIBROM); they were all women who had received a diagnosis of fibromyalgia (this is compulsory to gain membership of the association), according to the diagnostic criteria explained previously [30]. The patients had to meet the inclusion criteria and understand the purpose and design of the study over the phone. They were given an appointment at the fibromyalgia unit of the Rey Juan Carlos University, Madrid, where they first signed the informed consent and permission to tape the interviews. In this face-to-face session, participants filled out sociodemographic questions as well as the adaptation of the GPQ. Later, the interviewers started the focus groups by reviewing each of the nine questions–situations and discussing the extent to which they agreed with them and to also describe the meaning and experience of fatigue according to each situation. All the patients agreed to participate in the study, and there were no dropouts.

### 2.5. Data Collection

Data from all of the women were collected over a four-week period between February and March 2018. The three focus groups were composed of 8–9 participants (9 for Groups 1 and 2, and 8 for Group 3) and lasted, on average, 90 min. First, they completed the adaptation of GPQ, with nine fatigue-related situations (Table 1) that allowed topics relating to fatigue in fibromyalgia to emerge. Second, the participants were asked to mention everything that they experienced in relation to fatigue and for each situation.

The instructions given were the following: “In this questionnaire, daily life situations that may happen in your life are described. Read each one carefully and try to imagine being in that situation the best you can. They are hypothetical situations. It is possible you have never lived nor will live these situations (for example, if you cannot work or use a computer in your work). We ask of you to answer these imagining you are in that situation. Pay attention, it is not what you would do, but what you would think in that situation. Each situation is followed by a thought that a person can have in it. Therefore, you are then asked to indicate your degree of agreement with that thought, circling a number from 1 to 6. The greater the number, the more you agree with that thought. Remember, the question is not what you would do, but what you would think in that situation”.

After they indicated their degree of agreement with each thought, the interviewers asked the next follow-up questions concerning fatigue for each of the situations: (1) Regarding this particular situation, what is the meaning of fatigue for you? (2) Can you describe your experience of fatigue in this particular situation? These questions were guided to obtain information regarding specific topics of interest [22] and to get a more in-depth description and understanding of the experience of fatigue in FM [20]. Due to the cognitive impairment presented in FM patients (attention and concentration difficulties) [33,34,35], it is recommended to start with a structured guide, as this could avoid open and unstructured discourse and make sure we focus on the outcomes that were intended to be evaluated. Besides, the need to express the negative impact of the symptoms could inflate the actual experience, and the complaints could be influencing the group discussion situation [27]. As might be expected, during the course of the focus groups, having a guide was not an impediment for flexibility regarding other topics that could arise in addition to fatigue.

The interviews were conducted by LVF, SBR, and LSC and assisted by LGH and BMP, and they were recorded with a recording device inside a mobile phone. Later, all the audios were transcribed verbatim by DGC, who was free of any influence that could sway the transcription. After transcribing them, all the recordings were deleted from the phone to grant the participants anonymity. Overall, 269 min were recorded: 80 min for the first focus group, 94 for the second, and 95 for the third. The mean duration was 90 min. All groups were conducted at the fibromyalgia unit of the Rey Juan Carlos University, Madrid.

### 2.6. Data Analysis

Complete verbatim transcripts were produced for each group. Qualitative data were analyzed following a content thematic analysis using QRS Nvivo 10 software (QSR International, Burlington, MA, USA) [36]. Sample characteristics were analyzed by IBM SPSS 22 (IBM, Foster City, CA, USA). Under the supervision of CPP, MPM, and SLR, who have expertise in qualitative research and in fibromyalgia, LVF, LGH, and BPP divided the text into individual coding using the participant’s own words when possible after reading the transcripts. Following this process, these three authors (LVF, LGH, and BPP) created category schemes according to the proposed model [22]. The research team met every week to compare the content analysis categories, the emerging themes, and to discuss any issues that arose during the process. Since codes are not always mutually exclusive, a piece of text could be assigned to several codes [37]. Groups of codes that expressed the same ideas or phenomena were classified broadly into categories. In the case of differences of opinion, theme identification was decided by consensus. The final outcome was the identification of categories and subcategories that represented the experience of fatigue in the nine different situations that may occur in the daily lives of fibromyalgia patients.

### 2.7. Rigor

To ensure methodological rigor and to support the utility of the findings from this study, the authors followed the COREQ and SRQR guidelines [22,23] for qualitative research. In addition, selected criteria were applied to achieve study rigor, using specific strategies for each criterion derived from the seminal work of Shenton [38] and discussed in Vaismoradi et al. [39]. First, credibility refers to the respondent validation, or feedback, to establish that the data being collected reflect the perspectives of the participants. In applying this strategy, each data source was analyzed (triangulation). In addition, team meetings were carried out during which the analyses were compared and themes were identified. The process of collecting and analyzing the data concurrently also aided in checking and verifying the study findings. The second criterion applied was transferability, referring to the applicability in other contexts and descriptions of the study performed, providing details of the characteristics of researchers, participants, contexts, sampling strategies, and the data collection and analysis procedures. The third criterion used was dependability. This criterion emphasizes the need to define the study sample, specific enrollment criteria, and geographic area. Furthermore, an audit was carried out by an external researcher (in this study, there were three); this included an assessment of the study research protocol and suggestions for improving the study design. The last and fourth criterion to ensure the rigor of the study methodology was confirmability, which involved the team members maintaining neutrality with regard to the data. That is, the researchers were attentive to their interests, any bias, and/or motivations, not allowing these to influence the study findings and acting with an audit trail (maintaining a relatively transparent description of the research steps they took from the beginning of the research project through to the development and reporting of the findings) and reflexivity (systematic attention to the research process and what it was yielding throughout the study) [38,40].

### 2.8. Ethical Considerations

The study was performed according to accepted guidelines on ethical practice of the World Medical Association of Helsinki [41] and followed the Spanish Biomedical Research Act [42]. The study was approved by the Clinical Research Ethics Committee at the Rey Juan Carlos University (code: PI17/00858). All the participants signed informed consent forms and gave their permission to be recorded with a recording device inside a mobile phone verbatim, deleting them after transcription to grant the participants anonymity. In addition, every patient was assigned a numeric code.

## 3. Results

Twenty-six women with fibromyalgia were recruited. These women were mostly middle-aged (54.92 years; SD = 7.05; range = 41–70), and the diagnosis was made an average of 12 years ago (SD = 7.40). Concerning how they were diagnosed, most of the sample was diagnosed with fibromyalgia by the Rheumatology Unit of their referral hospital (73.07%). A large part of the participants had been dealing with pain for more than 26 years (34.6%) with an average of 22.33 years (SD = 12.98). Most of them were married or living with a partner (80.80%, *n* = 21), 7.70% (*n* = 2) were separated/divorced, 7.70% (*n* = 2) were widows, and only 3.80% (*n* = 1) were single. In relation to the employment status, 53.8% (*n* = 14) were working; of them, 26.90% were on temporary leave at the time of the study, 19.40% (*n* = 5) of the sample were housewives, and the rest were unemployed (11.50%, *n* = 3) or retired (11.50%, *n* = 3 due to pain, 3.80%, *n* = 1 due to other circumstances). Participants had, at least, primary studies (30.76%, *n* = 8), while most of them had secondary studies (50%, *n* = 13), and 19.40% (*n* = 5) had university studies. In relation to clinical variables, pain severity level was 6.53/10 (SD 3.28), 80.76% (*n* = 21) were using analgesics, 76.92% (*n* = 20) were using antidepressants and 65.38% (*n* = 17) were using muscle relaxants. All of them reported fatigue (100%, *n* = 26) and cognitive disturbance (100%, *n* = 26), and 80.76% (*n* = 21) reported depression. Table 2 shows a summary of the information regarding the demographic and clinical features of the sample.

### 3.1. Major Themes

Twenty-five themes were identified within the verbatim transcriptions of the participant’s conversations; these were categorized according to the ABC proposed model of fatigue in the nine different situations of the GPQ (Table 3). In particular, 5 antecedents, 13 behaviors (7 biological and 6 cognitive–emotional), and 7 consequences were categorized. Table 4 shows specific examples of the coding of all subcategories together with the descriptions of each of them. Table 5 shows the details of all the categories and subcategories identified in the discussion groups and the total references of the coding system. From the 25 subcategories, a total of 9 main themes were identified (one for the antecedents category, five for the behavior category—two biological and three cognitive–emotional, and three for the consequences).

First, the most prevalent theme within the antecedents’ category was the self-imposed duties, with a total of 58 references. Second, in the behaviors category, we found that muscle fatigue (26 references) and overwhelming feeling of tiredness (24 references) were the most prominent regarding the biological category, whereas within the cognitive–emotional category, we found difficulty thinking (30 references) as well as difficulty concentrating (23 references) and negative emotions (35 references). Lastly, in the consequences category, we found that lifestyle changes (27 references), affected everyday activities (12 references), and lack of motivation (7 references for the daily activities subcategory and 12 references for the social interactions subcategory) were the most outstanding themes. In addition, it is important to mention that during the analyzing and coding phase, we found some topics that were not included in the tentative model first stated but that are worth mentioning such as social support (3 references), resources (3 references), physical symptoms related to pain (9 references), and sexual difficulties (4 references).

#### 3.1.1. Self-Imposed Duties

Participants stated that work is very important to them; if faced with a hypothetical situation in which they must hand in a report, in spite of being tired and fatigued, they will still push themselves to finish it.

*“I have to do it no matter what.”*

*(P2, Group 1)*

*“If a report is to be finished or if a few people need to be called by tomorrow, I finish that report and call said people, I don’t go home without having done it.”*

*(P5, Group 1)*

That trend to tire oneself out is also present in the home and family context. Some participants pointed out that they give themselves strict deadlines to get their chores done, whereas others had children and said that they are obliged to take care of them and it cannot be postponed.

*“Everyone is focusing on work, but sometimes you’re home and there’s some things you can’t just leave undone.”*

*(P1, Group 1)*

*“If your child is crying you have to pick them up, and you have to pick them up.”*

*(P4, Group 1)*

*“You must finish ironing today, it has to be done no matter what, and nobody says that to me but myself.”*

*(P8, Group 1)*

#### 3.1.2. Muscle Fatigue

Participants claimed that muscle fatigue usually struck while they were busy doing something that required manual handling such as writing, drawing, coloring, sewing, or even driving.

*“Tingling or numbness in the hands.”*

*(P2, Group 2)*

*“I have to write a lot and my fingers get blocked, like my hands are clogged up and then I need to [gesticulates] do this, clench and unclench them until I can move them again.”*

*(P9, Group 1)*

*“Sometimes I’m sewing a purse with my kid’s old trousers and I spend 3 days doing it, because while I’m at it my hands become numb and I need to stop doing it.”*

*(P2, Group 2)*

*“I feel the same… I liked driving before… I could travel 300 or 400 km and now just 60 km make me tired. My hands and feet become numb and my head starts aching…”*

*(P3, Group 2)*

However, there are also cases in which they were doing relatively nothing and they were also overwhelmed by muscle fatigue.

*“As for me, it’s like numbness in the muscles, also pain, your muscles are clogged and muscle strength is lacking. Sometimes I’m on the sofa and the muscles start twitching on their own, like muscle spasms.”*

*(P2, Group 3)*

#### 3.1.3. Overwhelming Feeling of Tiredness

Participants seemed to be able to tell the difference between being just tired and truly fatigued or exhausted. Something interesting to point out is that they emphasized that while tiredness goes away with resting, this is not the case for fatigue.

*“It’s exhausting.”*

*(P5, Group 2)*

*“Some days I’ve been so exhausted from work that [I] said, ‘Okay, let’s take a nap,’ and then slept through all Friday evening and woke up directly on Saturday.”*

*(P6, Group 1)*

*“That tiredness, for me, is corporal and it’s more like… being exhausted, when you sleep and sleep and you can spend three days sleeping and you’re still tired. You even get tired just walking a little to go to the restroom.”*

*(P6, Group 1)*

#### 3.1.4. Difficulty Thinking

There seemed to be a consensus between participants that the difficulty thinking is characteristic of people with fibromyalgia; it is generally known as “fibrofog”. Said ‘fibrofog’ happened to them while doing something specific or just going somewhere.

*“You can’t think and you can’t hear, and everything’s gray—it’s like a mental fog.”*

*(P2, Group 3)*

*“At a cognitive level, it’s like you’re blocked at some point and you don’t understand anything anymore.”*

*(P5, Group 1)*

*“It’s like a mental fatigue.”*

*(P6, Group 1)*

*“It’s exhausting, because it’s like my cognition doesn’t respond, my thoughts don’t flow correctly and I can’t advance.”*

*(P4, Group 1)*

*“There was once a doctor who told me this is called fibrofog; we have so many thoughts because we want to encompass so many things that we get blocked and that’s all.”*

*(P4, Group 1)*

*“You get blocked by that fibrofog, even a simple question like two plus two leaves you blank with no answer, because your mind gets blocked.”*

*(P7, Group 2)*

*“Sometimes you don’t even know dates you had, all pictures seem the same…”*

*(P8, Group 2)*

*“You stay blank, just like that. It’s like that fog… it leaves you empty and you don’t know what to write or how to do it.”*

*(P2, Group 3)*

#### 3.1.5. Difficulty Concentrating

Participants usually described situations in which they were unable to focus on the task at hand, whether it be something work related such as accounting and finances or something for leisure such as reading a book.

*“Cognitive slowness.”*

*(P5, Group 1)*

*“I can’t focus my attention long enough, so I’m usually slow doing those things.”*

*(P9, Group 1)*

*“While I’m crocheting, I lose track of the holes and such and I need to undo it and the slowness is incredible.”*

*(P9, Group 1)*

*“Just reading once before I managed to grasp the meaning of everything, but now I need to read and reread it at least 3 or 4 times to understand the sentence.”*

*(P1, Group 3)*

*“I’m keeping the accounts of work and suddenly the numbers are like dancing and I can’t focus on them.”*

*(P6, Group 3)*

*“While I’m reading a novel, sometimes I just pause and ask myself, ‘What happened to this character now?’ because I lose track of the plot.”*

*(P2, Group 2)*

*“Before, I could read once what I was supposed to read and I could understand it, but now it’s like my mind gets blocked and I can’t seem to understand a thing… Maybe it’s just my mind.”*

*(P7, Group 2)*

*“When you’re reading that, fibrofog is also there, prevents me from reading because I just finished reading the third line and I can’t remember what the first line was about.”*

*(P1, Group 3)*

#### 3.1.6. Negative Emotions

Participants described feelings of agitation or restlessness because of the unpredictability of fatigue. They also feared surpassing their limits and being bedridden the next day.

*“It is fatigue, you feel it in your chest, like pressure, more like anxiety.”*

*(P1, Group 1)*

*“It leaves you restless.”*

*(P4, Group 1)*

*“You feel overwhelmed, antsy, and tired… leaves you with a feeling of impotence”.*

*(P1, Group 1)*

*“It’s despair.”*

*(P9, Group 1)*

*“Disquiet, impotence”.*

*(P1, Group 2)*

*“You manage your strength because you fear being unable to move the next day.”*

*(P1, Group 2)*

*“You feel impotence because you know you can’t go further.”*

*(P6, Group 2)*

#### 3.1.7. Lifestyle Changes

Participants emphasized that self-imposed duties had decreased over the years in favor of them having a better quality of life. Some examples of prioritizing their health over finishing their self-imposed tasks are shown below.

*“I’ve moved on, I learnt to leave things undone when needed.”*

*(P1, Group 2)*

*“I always took the car to wash, and now I tell my husband to help or to go himself.”*

*(P8, Group 1)*

*“I wasn’t always sure what was a necessity and what was self-imposed. Before, I was vacuuming daily and now not so much. Also, before I was always alone shopping and now I take my husband along to help.”*

*(P5, Group 1)*

*“If I’m too tired I sit on a bench and rest for a while before going home.”*

*(P3, Group 1)*

*“I’ve learnt not to clean the windows daily, some days I do some chores and some other day I do other chores, I don’t tire myself so much now.”*

*(P1, Group 2)*

*“Sometimes I organize chores and say, ‘Okay, tomorrow I need to be ironing shirts, then this other thing,’ and I plan beforehand so I don’t get overwhelmed with chores.”*

*(P9, Group 2)*

*“Years ago, I went shopping once a week, but now I go more often and to smaller shops instead of a big shopping center.”*

*(P1, Group 3)*

#### 3.1.8. Affected Everyday Activities

How fatigue affects not only strenuous physical activities but also cognitive tasks or simple self-care activities.

*“Fatigue is tiredness, it is the inability to do anything, total weakness, not having strength.”*

*(P6, Group 1)*

*“I get tired with any task, I don’t care if it is cleaning windows, and I feel frustrated because I know that I never finish anything.”*

*(P8, Group 2)*

*“It causes me a lot of fatigue doing things… for example, making the bed”.*

*(P5, Group 3)*

*“Every cleaning task and ironing is strenuous.”*

*(P3, Group 3)*

#### 3.1.9. Lack of Motivation for Social Interactions

Some participants said they were eager to spend time with people, but being in the situation left them tired, while other participants pointed out that they are already isolated because of fatigue and therefore have no desire to spend time with others; they seemed to be in despair regarding the idea of social life.

*“As the party goes on, you slowly turn off and start to feel tired and then you’re wishing they go already.”*

*(P6, Group 2)*

*“Slowly, we isolate ourselves, we have less social activity, because of tiredness, and it’s normal…”*

*(P5, Group 1)*

*“It leaves you exhausted and you prefer doing less and less, because fatigue leaves you totally depleted.”*

*(P2, Group 1)*

*“Sometimes you question if it’s worth it having a group of friends at all.”*

*(P8, Group 1)*

*“I like having people in the house, but then I start feeling antsy…”*

*(P6, Group 2)*

*“Yes, it leaves me restless, because I need to organize how many plates, glasses… and I start wondering how much left until they’re gone.”*

*(P7, Group 2)*

*“As the event goes by you slowly fade out, you’re like a burning candle that starts dimming low, and then you feel bad because you were so eager to be here and now you’re wishing to go home…”*

*(P4, Group 3)*

#### 3.1.10. Lack of Motivation for Daily Activities

Participants emphasized that daily chores were now more challenging and that they could not find the same strength or motivation to carry them out. In addition, there was one interesting point in their speech regarding technology not being advanced enough to help people with fibromyalgia.

*“I have a Roomba and sometimes I’m too lazy to crouch down and turn it on.”*

*(P4, Group 2)*

*“When you’re fatigued the first thing you lose is motivation, you don’t want to do anything, it’s like you’re flat tired.”*

*(P3, Group 2)*

*“Yes, but the point is having motivation to go shopping, it’s true they send it home to you later, but…”*

*(P1, Group 2)*

*“Just thinking I have to vacuum tires me, so I end up just sweeping the floor a little.”*

*(P2, Group 2)*

*“I think technology is not advanced enough to help people with fibromyalgia, like… Let’s see… I hope you understand me, but… devices, yes, they’re not fit for people like us, they’re made for people who are alright.”*

*(P6, Group 3)*

### 3.2. The 4 U’s Rule

In addition to the ABC model, and as something that emerged from the previous analysis, we found that patients also expressed four different characteristics that described their fatigue experience in the nine different situations of the GPQ. Detailed findings to support each of these fatigue descriptors are provided in Table 6, each with sample participant quotes.

#### 3.2.1. Unpredictable

The unpredictability of not only the fatigue, but also of its repercussions on life makes participants feel agitated or restless. They fear that normal tiredness can turn into fatigue and stop them from doing any daily activity (household, work, making phone calls, connecting on social media). This unpredictability makes them feel insecure and uncomfortable in social situations.

#### 3.2.2. Uncontrollable

Fatigue appears suddenly and without warning. This is a difficult consequence for patients, as in a normal day they are unable to ignore its intrusiveness, whilst they try to carry on with their different activities. Their body feels exhausted and makes it impossible for them to move or to rest. It also affects their emotions, making them feel anxious, desperate, and frustrated.

#### 3.2.3. Unseen

The subjective experience of fatigue sometimes makes it difficult to verbalize and to be seen. Patients verbalize that fatigue is not something that you can see in the body; it is invisible, and the exhaustion comes not only at a physical but also at a cognitive level.

#### 3.2.4. Unintelligible

Patients with fibromyalgia find their own illness emotionally distressing and difficult to understand. Social relationships can be affected due to lack of understanding from significant others or relationships can become weakened, for example with co-workers or people that are not particularly close. They feel not only misunderstood but also as if their credibility is in question.

## 4. Discussion

The present study aimed to identify and understand the variables involved in fatigue in nine different situations of the GPQ that may occur in the daily lives of women with fibromyalgia. According to the proposed model, nine major themes were identified, which are in agreement with previous literature [8,11,13,14].

First, our findings showed that self-imposed duties were the theme that reflected most references. This is unsurprising, since the nine situations were related with duties that are considered to be self-imposed (e.g., making a work report, cleaning windows). This variable has also been described in previous studies among women with fibromyalgia [43], specifically in qualitative studies. In this sense, Grape et al. [13] found that women with fibromyalgia described themselves as being vigorous and active people who set themselves high standards, living a life with busy schedules of domestic chores, family, and professional work [13]. Participants in this study stated that work is very important to them and that they have to finish it despite their tiredness and fatigue. This pattern of excess activity could be related to the “behavior peaks” that patients with fibromyalgia have: periods of high levels of activity in moments without symptoms and periods of low levels of activity before the appearance of pain or fatigue [44,45].

Second, according to the biological behavior of fatigue, we found that muscle fatigue and overwhelming feelings of tiredness were the most referenced. These variables were also included in the study by Humphrey et al. [11] and are part of their conceptual model about fibromyalgia. Other studies have highlighted that this characteristic in fibromyalgia, sometimes describing fatigue as paralyzing [13], sudden, and even uncontrollable [8]. Another variable worth mentioning in biological fatigue is the non-restful sleep, which is a theme found in previous research [8,11,13,14].

Third, in relation to the cognitive–emotional behavior of fatigue, we found that difficulty thinking, difficulty concentrating, and negative emotions were the most referenced. According to previous research, many people dealing with fibromyalgia have problems maintaining their attention on the task at hand; therefore, they have problems memorizing as well as remembering [46]. Another reported problem is in relation to keeping track of a story’s plot while reading or watching television [8]. Patients seem to have trouble making decisions and getting motivated [11]. It should be taken into account that negative emotions such as anger, along with other affective problems such as depression and anxiety, have also been shown to impact mental health and quality of life in fibromyalgia [47]. The role of affective processes in the etiology, course, and prognosis of fibromyalgia has been widely addressed in previous literature. The negative affect component of the disease has been discussed from different perspectives. On the one hand, psychopathological approaches suggest that fibromyalgia is itself an affective disorder, and on the other hand, there are biopsychosocial approaches in which emotion is considered as a determining factor (along with other factors) in the development of the disease [48]. It is clear that fibromyalgia affects the ability to properly function due to the presence of pain, fatigue, and other physical symptoms. Previous studies have reported the presence of anxious and depressive symptoms, along with the inability to experience positive emotions, and therefore psychological well-being [49,50]; this negative emotionality has been addressed in different studies due to its high comorbidity in fibromyalgia. These negative emotions have been described on numerous occasions as being associated to the severity of pain, or other symptoms [48,51], as well as a decrease in quality of life [52].

Fourth, when identifying the consequences, we found that lifestyle changes were the most referenced. It has often been reported that FM patients suffer many changes in their lives due to the functional limitations this condition produces for the patients [53,54]. Grape et al. [13] found that people eventually integrate fatigue in their lives to adapt to their new situation, adjusting their energy levels for that day and learning to identify when to stop and rest. We found that those lifestyle changes included asking for help and delegating some tasks to their family members, such as husbands or older children [13]. Social life also suffers the consequences related to fibromyalgia; this includes not only social interactions but also the decreases the motivation to engage in them. It has been reported previously that fatigue reduces social life for fibromyalgia patients and that it has an effect upon the relationships with their families and friends, as they interpret fatigue as laziness [8]. Another study supporting this conclusion adds that the interpretation of the social event is key in predicting fatigue levels, which are lower if the social relationships are considered positive by the patient [12]. In our study, the lack of motivation is not only associated to social events but also to daily activities, which is a finding similar to that of the study by Humphrey et al. [11].

Furthermore, four additional themes were identified besides the ones proposed in the model. First, social support was identified as a positive lifestyle change for people with fibromyalgia, in the form of receiving help from their families. Other research has shown that social support is an important mechanism for coping with the experience of fibromyalgia [55], and it is considered a mediator of the relationship between role strains and marital satisfaction in husbands of fibromyalgia women [56]. Second, the fibromyalgia patients referred to the different strategies for pain and fatigue management used by them, such as physical exercise, using a coloring book, etc. Distracting from the pain is a particularly helpful strategy, and it is possible to learn or improve this skill through health-promoting courses based on cognitive behavioral therapy (CBT) [57]. Third, physical symptoms related to pain were mentioned in the discussion group when speaking of the somatic symptoms as a whole, such as headaches, muscular tension, neck pain and strain, etc. Specifically, in relation to symptoms such as headaches, which are comorbid with fibromyalgia [58], further evidence has been found that oxidative stress correlates with headache symptoms in fibromyalgia patients, therefore supporting the dysfunction of the HPA [59]. Finally, sexual difficulties was an emergent theme in relation to “lack of motivation for daily activities”. Previous studies have identified sexual problems and dysfunctions in both female and male population with fibromyalgia [60,61].

Throughout this article, the importance of fatigue in fibromyalgia has been highlighted, and we suggest that continuing study of this phenomenon is of great interest if we want better treatment and understanding of this condition. However, pain also needs to remain a focus, as our patients are never able to escape it, either. Even when not asking about pain, it is a theme that appears (physical symptoms) in the participant’s narratives; we are not debating whether pain is more important than fatigue or vice versa, but stating that both are important and we should ignore neither.

On the one hand, this study has been able to support the findings by Eilertsen et al. [8] in relation to fatigue not being eased with the usual management strategies for tiredness, as participants mentioned that their fatigue is not relieved by sleeping or resting [8]. On the other hand, we were unable to confirm any of the biological explanations for fatigue—HPA dysfunction—as we did not take any biological measures; however, stress was found to be one of the antecedents of fatigue in fibromyalgia. As stress has been pointed out as one of the causes of HPA dysfunction, our data would be in support of the results found by Riva et al. [9] and by Mahdi et al. [10]. Our study has been able to underline that stress is of great importance in fatigue for fibromyalgia patients; this should encourage the implementation of stress management treatments, with the aim of preventing fatigue from affecting these patients too harshly.

Finally, with our model, the one by Humphrey is complemented and it allows a better understanding of fatigue. It is essential to describe this symptom, as awareness needs to be raised in relation to its triggers and effects on patients. As an addition to the study by Humphrey et al., our findings have shown that it is possible to conceptualize the experience of fatigue among FM patients following, what we have called, the 4 U’s Rule model. The patients clearly expressed that these four characteristics of the model were both specific and differentiating in relation to fatigue: Unpredictable, Uncontrollable, Unseen, and Unintelligible. The unpredictability of the nature of not only the fatigue, but also of its repercussions in life; the uncontrollability of the symptom as it appears suddenly; the subjective experience that sometimes makes it complicated to verbalize and be seen; and the unintelligibility and lack of understanding.

This study presents a number of limitations. First, the participants included in the study belonged to a fibromyalgia association, and although all received the diagnosis following the diagnostic criteria established by the Spanish Ministry of Health and the 1990 guidelines of the American College of Rheumatology [31], our results cannot be extrapolated to the whole population with FM. The fact that this study was conducted in Spain—with its specific social and cultural characteristics in terms of its health care delivery system and the organization of fibromyalgia associations by provinces—may also have influenced the results obtained. However, we consider that they can most likely be applied to other contexts where patients have similar characteristics [3,4]. Second, the focus groups were guided with a structured format for nine specific situations, and the questions were asked in relation to these. In this sense, information may have been lost in relation to exploring or understanding the patients’ own perspectives, experiences, thoughts, and perceptions, and a more open structure would have allowed us to obtain more genuine content. However, we have learnt from previous experience [27] that the need for these women to express the negative impact of the disease can inflate the real experience of the symptoms. Third, the theme that appeared the most was self-imposed duties, which is probably because the nine situations were guided to specific thoughts about this kind of task. Although previous studies have described the busy schedules related with daily duties in patients with FM [13,43], exploring other themes would deepen our knowledge relating to their thoughts in other types of situations.

These findings may help achieve improvements in future interventions. As it has been mentioned before [6,27], fatigue (along with pain) is the most important behavioral belief that generates physical and psychological discomfort. This is why interventions that not only manage pain but also manage fatigue are crucial for these patients.

## 5. Conclusions

Our findings suggest that in addition to pain, fatigue is an important symptom in fibromyalgia, especially considering situations that may occur in patients’ daily lives, such as housework, working, or organizing meetings. However, little is known about how fatigue is experienced by fibromyalgia patients in these situations. The proposed ABC model shows elements that emerged from their fatigue experience, which can also be summarized in four adjectives: Unpredictable, Uncontrollable, Unseen, and Unintelligible for oneself and for others. Identifying these characteristics may help manage fatigue with specific strategies within interdisciplinary interventions to decrease the impact of this symptom in fibromyalgia patients.

## Figures and Tables

**Table 1:** Situations in the Goal Pursuit Questionnaire (GPQ) ^1^ for focus groups.

**Situation 1:** You are making a work report, either typing or writing it by hand. The fatigue is increasing with time. It is expected of you to finish that report today.
You think: It is more important to lower my fatigue now than to finish this report.
**Situation 2:** You are cleaning the windows. As you clean, you become more fatigued. It is expected of you to finish cleaning the windows today.
You think: It is more important to lower my fatigue now than to clean the windows.
**Situation 3:** You are accounting for income and expenses. After two hours, the sum still does not add up and you are feeling more and more reluctant to finish it. It is expected of you to finish the calculations today.
You think: It is more important to lower my fatigue now than to finish the calculations correctly.
**Situation 4:** You are carrying the shopping bags. After a while, you are fatigued. It is expected of you to finish shopping today.
You think: It is more important to lower my fatigue now than to finish shopping.
**Situation 5:** You are vacuuming your home. While you are doing it, you start feeling fatigued. It is expected of you to finish cleaning your home today.
You think: It is more important to lower my fatigue now than to clean your home.
**Situation 6:** You are making a picture album either by hand or on the computer. As you are doing it, you start feeling fatigued. It is expected of you to finish the album today.
You think: It is more important to lower my fatigue now than to finish the album.
**Situation 7:** You need to sew something. As you are sewing you start feeling more and more fatigued. It is expected of you to finish sewing today.
You think: It is more important to lower my fatigue now than to finish sewing.
**Situation 8:** You are washing your car. As you are washing it, you start feeling more and more fatigued. It is expected of you to finish washing your car today.
You think: It is more important to lower my fatigue now than to finish washing my car.
**Situation 9:** You call some people to organize a date for a meeting. Due to the numerous calls you need to make, you are feeling more and more fatigued with each passing call. It is expected of you to finish arranging the meeting date today.
You think: It is more important to lower my fatigue now than to arrange the meeting date.

^1^ GPQ: Goal Pursuit Questionnaire.

**Table 2:** Demographic and clinical features of the female participants with fibromyalgia.

	*n*	%	Mean	SD
**Age**			54.92	7.05
**Diagnosis year**			12	7.40
**Year Pain Began**			22.33	12.98
**Pain Severity (0–10)**			6.53	3.28
**Professional who Diagnosed**				
Rheumatology	19	73.07		
Traumatology	2	7.70		
Primary Care	4	15.38		
Others	1	3.80		
**Civil Status**				
Married	21	80.80		
Separated	2	7.70		
Widow	2	7.70		
Single	1	3.80		
**Employment Status**				
Employed	14	53.80		
Housewife	5	19.40		
Retired due to Pain	3	11.50		
Unemployed	3	11.50		
Retired Due to Other Circumstances	1	3.80		
**Education Level**				
Primary Studies	8	30.76		
Secondary Studies	13	50		
University Studies	5	19.40		
**Medication Use**				
Analgesics	21	80.76		
Antidepressants	19	76.92		
Muscle relaxants	17	65.38		
No Medication	3	11.50		

**Table 3:** The ABC (Antecedents–Behaviors–Consequences) proposed model of fatigue in fibromyalgia in the different situations of the GPQ.

Antecedents	Behaviors	Consequences
Catastrophism.	*Biological Fatigue:*	Affected Everyday Activities.
	Lack of Energy.	
	Overwhelming Feeling of Tiredness.	Affected Social Interactions.
	Non-Restful Sleep.	
	Not Proportional to Effort Exerted.	Lack of Motivation for Daily Activities.
	Feeling Weakness/Heaviness.	
	Muscle Fatigue.	Lack of Motivation for Social Interactions.
	Difficulty–Slowness Doing Things.	
		Lower Quality of Sleep.
	*Cognitive–Emotional Fatigue:*	
	Difficulty Concentrating.	Lifestyle Changes.
	Difficulty Remembering.	
Busy Schedule.	Difficulty Making Decisions.	Problems in Affectivity.
Demanding Social Interactions.	Difficulty Thinking.	
Family.	Difficulty Getting Motivated.	
Self-Imposed Duties.	Negative Emotions.	

**Table 4:** Categories, subcategories, descriptions, and examples of the coding.

Category	Subcategory	Description	Example for Coding
**Antecedents**	**Catastrophism**	Negative vision of the world, which is seen as menacing [14].	*“Even if I am in bed I am still exhausted; I can’t move my arms, I can’t move my legs, I can’t move my body. My worst nightmare is being unable to walk because of this.” (P9, G1)*
	**Busy Schedule**	Women with exacting standards about how they should lead their lives, filling their schedule with domestic chores, family care, and professional work [13].	*“Today I went shopping, needed to pick up my kids from school, needed to get lunch done…I’m just super tired.” (P2, G1).*
	**Demanding Social Interactions**	A negative appraisal of relationship engagement [12].	*“Your children think you’re below them, But no, not if I can’t, I can’t. Now everything that others demand from you, they demand constantly.” (P7, G1).*
	**Family**	Irritation or frustration among their family members, lack of understanding from them [8].	*“You try and try and still it seems wrong to them.” (P6, G3).*
	**Self-Imposed Duties**	Continue with work and home duties at the cost of her own health and well being [14].	*“If a report is to be finished or if a few people need to be called by tomorrow, I finish that report and call said people, I don’t go home without having done it.” (P3, G3).*
**Biological Behaviours**	**Lack of Energy**	Low strength or vitality, having no energy to do things [11].	*“Sometimes I say, ‘I’m done’ and then save up my energy.” (P9, G1).*
	**Overwhelming Feeling of Tiredness**	Tiredness that is persistent, unpredictable, and graver than normal tiredness [11].	*“Some days, I’ve been so exhausted from work that [I] said ‘Okay, let’s take a nap,’ and then slept through all Friday evening and woke up directly on Saturday.” (P6, G1).*
	**Non-Restful Sleep**	Feeling tired even after a good night’s sleep [11].	*“It doesn’t go away, even if you sleep or rest, you’re still tired.” (P5, G2).*
	**Not Proportional to Effort Exerted**	Being exhausted after doing hardly anything or becoming very tired doing very little [11].	*“You just walk a little to get to the bathroom and you’re already exhausted.” (P3, G3).*
	**Feeling Weakness/**	A subjective sensation of the body being heavier or being weaker [11].	*“You feel the heaviness, you feel crushed.” (P3, G1).*
	**Heaviness**		
	**Muscle Fatigue**	Decreased ability from the muscles to perform [11].	*“I have to write a lot and my fingers get blocked, like my hands are clogged up…” (P4, G3).*
	**Difficulty–**	Slowness and/or difficulty doing things [11].	*“I could make the bed in the blink of an eye, and now it’s 10 min…” (P9, G2).*
	**Slowly**		
	**Doing Things**		
	**Difficulty**	Difficulty focusing on reading or engaging in a conversation [8].	*“I’m keeping the accounts of work and suddenly the numbers are like dancing and I can’t focus on them.” (P4, G3).*
	**Concentrating**		
	**Difficulty Remembering**	Difficulty to remember dates or what to buy [8].	*“Sometimes I don’t remember which medication I’ve taken.” (P7, G2).*
	**Difficulty Making Decisions**	Insecurity that fatigue might strike again because of its unpredictability [8]	*“Making plans is difficult, you don’t know when you’re going to feel fatigued.” (P2, G1).*
	**Difficulty Thinking**	Inability to think clearly [11].	*“It’s like a mental fog.” (P1, G1).*
	**Difficulty Getting Motivated**	Lack of enthusiasm they need to overcome in order to do things [11].	*“You feel impotence and don’t want to continue doing it” (P3, G3).*
	**Negative Emotions**	Emotions such as despair, fear, anxiety…	*“You feel desperate, it’s real despair.” (P4, G2).*
**Consequences**	**Affected Everyday Activities**	Such as strenuous physical activities (e.g., playing sports, yard work), cognitive tasks (e.g., paying bills) and simple self-care activities (e.g., pouring a cup of coffee, getting dressed) [11].	*“Every task is tiring to me, be it cleaning the windows or vacuuming.” (P6, G2).*
	**Affected Social Interactions**	Unpleasant social situations that resulted from the unpredictable nature of fatigue [8].	*“It’s exhausting, you get tired calling people and explaining why…” (P5, G1).*
	**Lack of Motivation for Daily Activities**	Perceived as more demanding. They tried to perform regular household chores, but often found that they could not manage to finish the work they started [8].	*“Yes, but the point is having motivation to go shopping, it’s true they send it home to you later, but…” (P3, G2).*
	**Lack of Motivation for Social Interactions**	Social withdrawal as a shelter against unpleasant social situations that increased stress [8].	*“Slowly, we isolate ourselves, we have less social activity, because of tiredness, and it’s normal…” (P5, G1).*
	**Lower Quality of Sleep**	They often wake up in the night or have trouble falling asleep because of pain and/or fatigue. They suffer from uncontrollable and sudden sleepiness; they have noticed an increased need to sleep [8].	*“I only sleep like a couple hours daily…” (P6, G1).*
	**Lifestyle Changes**	Changes in daily activities and priorities [8]. In addition, changes in their social networks [14].	*“Years ago, I went shopping once a week, but now I go more often and to smaller shops instead of a big shopping center.” (P1, G3).*
	**Problems in Affectivity**	Fatigue made them enjoy social activities less and increased their stress [12]. Strained family relationship [8].	*“Sometimes I’m cleaning the kitchen and I feel like I can’t go on, then I start crying a whole lot…” (P7, G3).*

**Table 5:** Total references of the coding system.

Category	Name	Number of References
**Antecedents**	Catastrophism	9
	Busy Schedule	2
	Demanding Social Interactions	7
	Family	6
	Self-Imposed Duties	58
**Behaviours**	Lack of Energy	20
	Overwhelming Feeling of Tiredness	24
	Non-Restful Sleep	4
	Not Proportional to Effort Exerted	3
	Feeling Weakness/Heaviness	16
	Muscle Fatigue	26
	Difficulty–Slowness Doing Things	6
	Difficulty Concentrating	23
	Difficulty Remembering	19
	Difficulty Making Decisions	6
	Difficulty Thinking	30
	Difficulty Getting Motivated	1
	Negative Emotions	35
**Consequences**	Affected Everyday Activities	12
	Affected Social Interactions	7
	Lack of Motivation for Daily Activities	7
	Lack of Motivation for Social Interactions	12
	Lower Quality of Sleep	3
	Lifestyle Changes	27
	Problems in Affectivity	1
**Others**	External Support	3
	Resources	3
	Physical Symptoms	9
	Sex	4

**Table 6:** Characteristics, descriptions, and examples for the coding of the 4U’s rule.

Characteristic	Description	Example of Coding
Unpredictable	The unpredictability of not only fatigue, but also of its repercussions on life	“I was exhausted doing housework that I said never again.” (G2, P3)
		“With my tongue hanging out, I try to finish my work as well as possible. Then I end up crumbling and what do I do? Well, I lie down.” (G3, P8)
		“What fatigue is doing is that little by little we are becoming isolated, we have less social activity because that wears you out and you prefer to do less. You meet fewer times because of fatigue, because it exhausts you.” (G1, P5)
		“For me, what to do… I am so exhausted about… making calls and all that like following a WhatsApp group.” (G1, P7)
		“You have to do the report no matter what… so even if tiredness appears or you are fatigued, you have to finish it.” (G1, P2)
		“Because when I had the responsibility to work, I would go out at two in the morning and drag my body until two in the morning… and the next day, I couldn’t even get up.” (G2, P8)
Uncontrollable	Symptom appears suddenly	“Although I am in bed, I am still exhausted; I can’t move my arms, I can’t move my legs, I can’t move my body…” (G1, P9)
		“Many times we do not know how far we can go until we really fall. As I say to my husband: ‘I can’t take it anymore,’ but when I say, ‘I can’t take it anymore,’ I mean I had stopped being able to a long time ago.” (G2, P8)
		“When I think that I have to do something is when I can’t, I start to get nervous and I know fatigue is coming.” (G2, P3)
		“You’re there both with fatigue and without fatigue… you throw yourself head long, which is what she says.” (G3, P6)
		“I am exhausted right now. My motor won’t start again. It doesn’t matter if I lie down.” (G1, P5)
		“It is a brutal muscular exhaustion that just appears.” (G1, P7)
Unseen	The subjective experience that sometimes makes it difficult to verbalized and to be seen	“I mean sometimes I feel like I’ve aged 20 or 25 years in a matter of 2 years. It is as if my body is turning off due to the fatigue.” (G2, P5)
		“It’s not just the physical fatigue that bothers you, but the mental one, too.” (G2, P8)
		“As the event goes by you are exhausting, you are fading, you are reluctant… physically, mentally… It is like a candle, it is consumed and it is extinguished.” (G2, P6)
		“Fatigue is more physical for me and exhaustion is when you sleep and you sleep and you sleep… You can spend three days sleeping and you are still tired.” (G1, P6)
		“I can’t pay as much attention because of the fatigue, I mean it makes me cognitively slow.” (G1, P5)
		“You start reading and you have finished a paragraph and it gets foggy. By the third time you are going to start reading you no longer know where to start.” (G3, P2)
		“You go blank and you don’t know what you have to put or what you don’t have to put or how you have to put it.” (G3, P2)
Unintelligible	Lack of understanding	“It happens to me many times… to speak, I think of something, I am going to say it and I just can’t get out what I want to say, and I say to myself: ‘Am I stupid or what is happening to me?’” (G2, P6)
		“The truth that also happens to me. I go to any store and I have to choose between a lot of products and I become blocked automatically and people are surprised.” (G3, P5)
		“At the cognitive level, there comes a time when there is a blockage and they do not understand it. I mean, uh… it doesn’t matter, it’s that they don’t understand it and they don’t see it…“ (G1, P5)
		“Your children, who think you are beneath them, but if I can’t, I can’t. To everything that others demand of you. They demand a lot from you…” (G1, P7)
		“because if there is no environment that supports you, then you just have to f***ing do it”. (G1, P6)
		“It is expected of you that you have to finish it, and without excuses you have to finish it, above all.” (G1, P7).
